# Regulation of ROS signaling by TIGAR induces cancer-modulating responses in the tumor microenvironment

**DOI:** 10.1073/pnas.2416076121

**Published:** 2024-12-05

**Authors:** Eric C. Cheung, Douglas Strathdee, David Stevenson, Jack Coomes, Karen Blyth, Karen H. Vousden

**Affiliations:** ^a^The Francis Crick Institute, London NW1 1AT, United Kingdom; ^b^Cancer Research UK Scotland Institute Scotland Institute, Glasgow G61 1BD, Scotland; ^c^School of Cancer Sciences, University of Glasgow, Glasgow G61 1QH, Scotland

**Keywords:** oxidative stress, pancreatic cancer, tumor microenvironment

## Abstract

The consequences of reactive oxygen species (ROS) in cancer cells are complex and have been shown to both promote and retard tumorigenesis in different models. In mouse models of pancreatic ductal adenocarcinoma (PDAC), loss of the antioxidant defense gene *Tigar* results in both a reduction in the development of early pancreatic intraepithelial neoplasia and an increase in invasive and metastatic capacity, accompanied by decreased survival of mice lacking pancreatic TIGAR. We previously demonstrated that increased ROS following loss of TIGAR promotes various cancer cell–intrinsic changes that contribute to metastatic capacity, including epithelial to mesenchymal transition, enhanced migration and invasion, and an increase in ERK signaling. In this study, we show that pancreatic overexpression of TIGAR decreases metastatic capacity and migratory phenotypes in an aggressive model of PDAC, consistent with the concept that dynamic modulation of TIGAR in PDAC contributes to the development and progression of these tumors. Using TIGAR deficient and overexpressing mouse models, we find that the impact of modulation of TIGAR and ROS in PDAC cells also has a profound effect on the normal stromal cells surrounding the tumor. Loss of TIGAR promotes the production of cytokines by cancer cells that induce changes in the surrounding fibroblasts to adopt a tumor-supportive phenotype. Furthermore, these cytokines also attract macrophages that support PDAC dissemination and metastasis. Taken together our work shows that TIGAR-modulated ROS in PDAC can control cell intrinsic and extrinsic changes to impact tumor aggression.

## Significance

Reactive oxygen species (ROS) in cancer cells can both promote and suppress malignant progression, depending on the tumor type and stage of cancer development. TIGAR deficiency, which leads to more ROS, increases metastasis in a mouse model of pancreatic cancer, and we show here that overexpression of TIGAR (resulting in less ROS) decreases tumor invasiveness. We find that ROS levels in the cancer cells impact their interaction with surrounding normal stromal cells, with higher ROS in cancers that have lost TIGAR inducing a more tumor-supportive behavior of surrounding fibroblasts and macrophages. Our study shows that in addition to affecting the behavior of the cancer cell, TIGAR and ROS can impact surrounding cells to modulate tumor aggression.

The development and progression of cancer are controlled by numerous cell intrinsic and extrinsic factors. Many events that accompany metastasis—such as activation of oncogenes, loss of stromal support, and circulation in blood or lymph—induce increased oxidative stress that can cause tumor cell death and limit the success of metastatic colonization (1). However, reactive oxygen species (ROS) are important signaling molecules that can drive tumor proliferation and invasion (2, 3). These diverse ROS activities lead to difficulties in predicting the outcomes of therapies involving ROS, as ROS can either promote (4–6) or impede (7–9) tumor growth. TIGAR is a protein that can augment antioxidant capacity (10), and while TIGAR is not required for normal growth and development, it is overexpressed in many human cancers (9, 11, 12). In mouse models, TIGAR can support the development of intestinal (9) and lymphoid tumors (13) and in a model of pancreatic ductal adenocarcinoma (PDAC), loss of TIGAR limited the development of pancreatic premalignancies. However, loss of TIGAR increased metastasis of these PDACs to the lung. This effect is accompanied by an epithelial to mesenchymal transition of the PDAC cells, enhanced migration, and ERK signaling that is dependent on increased ROS (4). In mouse and human PDAC, levels of TIGAR expression oscillate as tumors progress, consistent with a differential role of ROS regulation at different stages of malignant development.

While tumor intrinsic changes play an important role in controlling cancer behavior, the stromal cells surrounding the tumor are also critically important. Substantial cross talk exists between tumor and stromal cells, with evidence that cancer cells can reprogram surrounding fibroblasts, adipocytes, endothelial, and immune cells to become tumor supportive (14). Previous studies have shown that the ROS status of both cancer cells and stromal cells can impact these interactions. For example, ROS in both tumor and endothelial cells can enhance angiogenesis to provide tumors with a blood supply for nutrient access and dissemination (15–17). Increased ROS in fibroblasts can convert these cells into tumor-supportive cancer-associated fibroblasts (CAFs) (18–21). Many immune cells are regulated by ROS, with evidence that ROS can be necessary for function but—when excessive—is also limiting for survival and efficiency of both pro- and antitumoral immune cells (22, 23). For example, ROS produced by cancer cells can recruit and support tumor-associated macrophages while also limiting the recruitment of potentially antitumoral T-cells (24–29).

We have used modulation of TIGAR in genetically engineered PDAC mouse models to investigate the impact of tumor-derived ROS on stromal cells in the tumor microenvironment (TME). Consistent with our previous studies showing that TIGAR loss promotes increased lung metastasis, we find that elevated TIGAR expression retards PDAC metastasis to the lung and decreases the migratory phenotype of the tumor cells in vitro. We also show that modulation of TIGAR expression and ROS levels promotes cytokine production by the cancer cells, driving the acquisition of cancer-supporting phenotypes in tumor-associated fibroblasts and myeloid cells. The role of stromal cells in modulating the invasiveness of tumor cells will be important to consider when considering the response to ROS regulation.

## Results

### Overexpression of TIGAR Limits PDAC Metastasis to the Lung.

We previously used an established poorly metastatic murine model of PDAC driven by mutation in KRAS and loss of p53 (termed KFC) to generate mice that also carried pancreatic loss of TIGAR (termed KFC-KO mice) (4). In this model, we showed that the increase in ROS following TIGAR loss promoted enhanced metastasis to the lung. To understand whether sustained overexpression of TIGAR affects PDAC progression, we generated a mouse conditionally overexpressing TIGAR (*SI Appendix*, Fig. S1*A*). These mice were crossed into a PDAC model carrying mutations in both KRAS and p53 (termed KPC), which shows significantly more metastases than the KFC model (30). In this KPC model, we used pancreas-specific p48-Cre (31) instead of Pdx1-Cre that was used in the KFC model, to limit the appearance of background tumors (32). The mice carrying pancreas-specific expression of the TIGAR transgene (termed KPC-Tg mice) showed clearly elevated TIGAR expression in PDAC compared to tumors from control KPC mice (*SI Appendix*, Fig. S1*B*). While TIGAR levels were variable in other normal tissues, their relative expression was similar in KPC and KPC-Tg mice (*SI Appendix*, Fig. S1*B*). High levels of TIGAR expression in muscle have been described previously (33) and shown to support oxidative stress and mitochondrial function.

**Fig. 1.**
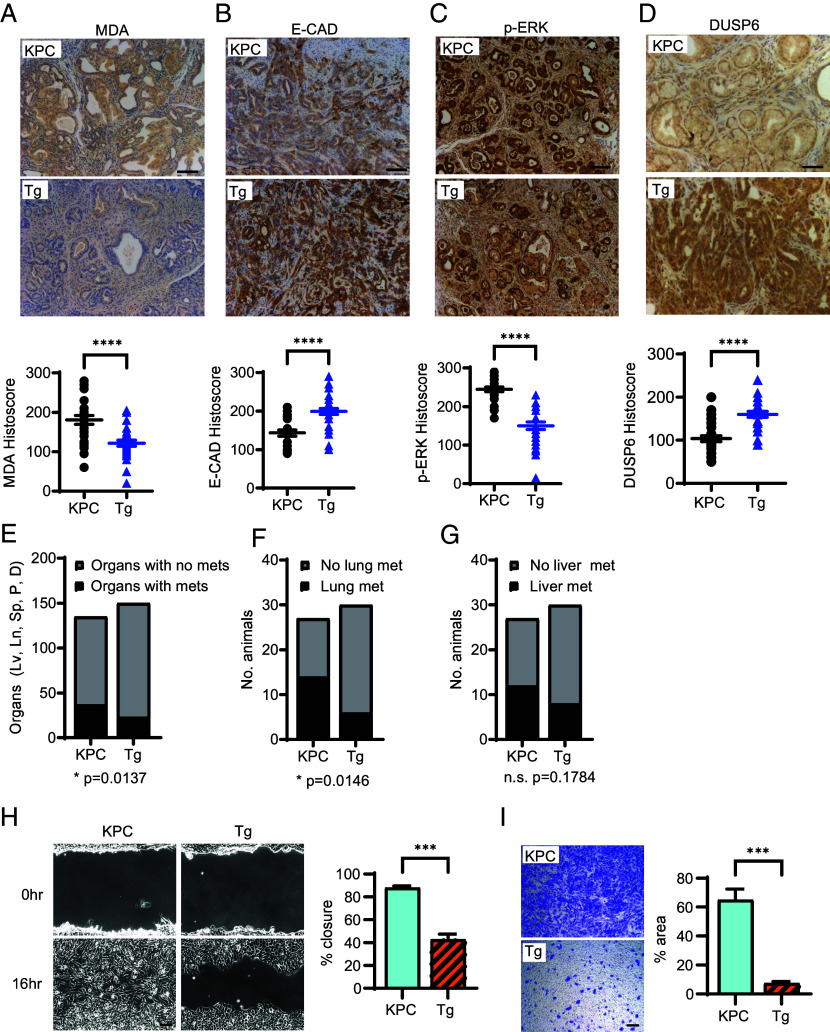
TIGAR overexpression in KPC PDAC model reduced lung metastasis and migration. (*A*–*D*) Immunohistochemistry and quantification of (*A*) MDA (*B*) E-CAD (*C*) p-ERK (*D*) DUSP6 expression in PDAC from KPC and KPC-Tg mice. Each data point represents values obtained from an individual animal. KPC: n = 27, KPC-Tg: n = 30 (*E*) Percentage of organs (liver:Lv, lung:Ln, spleen:Sp, peritoneum:P, diaphragm:D) with metastasis in KPC and KPC-Tg mice. (*F*) Number of mice with lung metastasis. (*G*) Number of mice with liver metastasis. (*H*) Wound-healing of KPC and KPC-Tg cells. (*I*) Transwell migration of KPC and KPC-Tg cells. Data in (*A*–*D* and *H*, *I*) were analyzed by two-tailed Student’s t test. Data in (*E*–*G*) were analyzed by Fisher's exact test. (*H* and *I*) n = 3 independent cell lines for KPC and n = 4 independent cell lines for KPC-Tg. Error bars represent mean ± SEM. *** *P* < 0.001; **** *P* < 0.0001(Scale bar, 100 μm.)

Corresponding to the increase in TIGAR expression (*SI Appendix*, Fig. S1*B*), PDAC tumors that arose in the KPC-Tg mice showed a decrease in oxidative stress, as measured by MDA staining of lipid peroxidation (Fig. 1*A*). In previous studies, increased ROS resulting from loss of TIGAR in KFC-KO tumors was shown to enhance the acquisition of a mesenchymal phenotype and increased ERK signaling. Here, we found that TIGAR overexpressing KPC-Tg tumors retained higher expression of E-Cadherin (an epithelial marker, Fig. 1*B*), lower levels of ERK activation (as indicated by less phosphorylated ERK, Fig. 1*C*), and increased expression of DUSP6 (a phosphatase that dephosphorylates and so inactivates ERK, Fig. 1*D*) compared to KPC tumors. TIGAR overexpressing PDAC tumors metastasized to various organs, including lung, peritoneum, spleen, and liver, indicated by staining with the pancreatic marker CK19 in these organs (*SI Appendix*, Fig. S1*C*). Although overexpression of TIGAR did not significantly alter the number of mice with evidence of metastases compared to control mice (*SI Appendix*, Fig. S1*D*), KPC-Tg mice maintained a significantly lower metastatic burden, as indicated by the total number of organs containing metastases (Fig. 1*E*). Consistent with previous work showing loss of TIGAR selectively enhanced lung metastasis (4), we found that overexpression of TIGAR limited invasion to the lung but did not significantly influence metastasis to the liver (Fig. 1 *F* and *G*). However, TIGAR overexpression did not impact survival rates, which were similar in both KPC and KPC-Tg mice (*SI Appendix*, Fig. S1*E*). Further characterization of cell lines derived from tumors showed an increase of E-cadherin and DUSP6 and decrease in ERK phosphorylation in KPC-Tg compared to KPC cells (*SI Appendix*, Fig. S1*F*). The cells derived from KPC-Tg tumors also retained a more epithelial appearance than KPC tumor cells (*SI Appendix*, Fig. S1*G*) and were less migratory in both wound healing and transwell assays (Fig. 1 *H* and *I*). Overexpression of TIGAR in metastatic pancreatic tumors therefore served to limit ROS, migration, and metastasis to the lung, consistent with the phenotypes observed in TIGAR null PDAC (4).

**Fig. 2.**
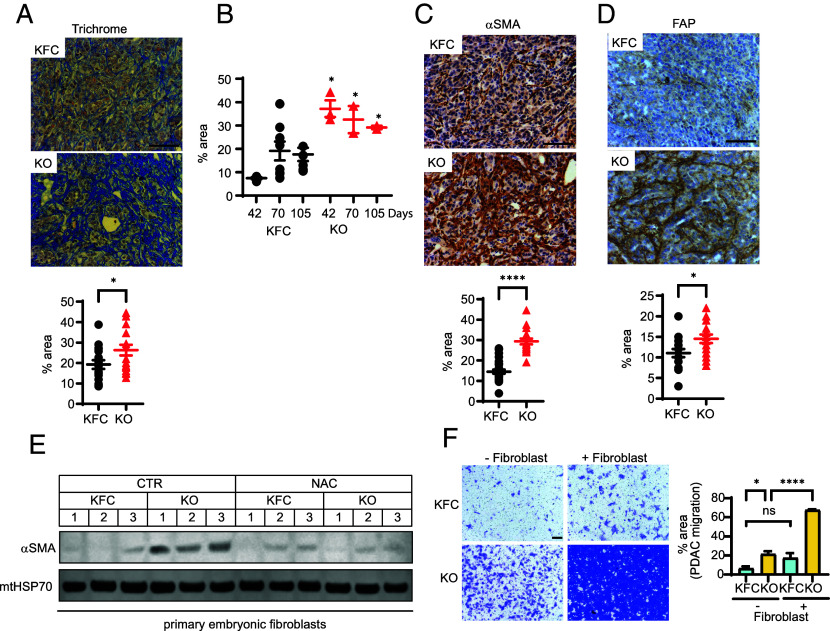
Alteration in tumor associated fibroblasts in TIGAR deficient PDAC tumors. (*A*) Trichrome staining and quantification of PDAC from KFC and KFC-KO mice. (*B*) Quantification of trichrome staining of KFC and KFC-KO tumors collected from mice at the indicated age (days). (*C*–*D*) Immunohistochemistry and the quantification of (*C*) ⍺SMA and (*D*) FAP expression of PDAC from KFC and KFC-KO mice. (*E*) Western blot analysis of ⍺SMA level in mouse primary embryonic fibroblasts cocultured with KFC and KFC-KO cell lines treated with (NAC) and without (CTR) N-acetyl cysteine (1 mM). Fibroblasts from coculture with three independent PDAC cell lines are shown. Loading control is mtHSP70. (*F*) Transwell migration of KFC and KFC-KO cells in the presence (+) or absence (−) of mouse primary embryonic fibroblasts. N = 3 independent KFC and KFC-KO cell lines. Data in (*A*, *C* and *D*) were analyzed by two-tailed Student’s t test. Error bars represent mean ± SEM. Data in (*B* and *F*) were analyzed by one-way ANOVA with Tukey post hoc test. Each data point in (*A*–*D* and *F*) represents values obtained from an individual animal, n = 16-17. * *P* < 0.05; ** *P* < 0.01; *** *P* < 0.001; **** *P* < 0.0001. (Scale bar, 100 μm.)

To explore the effect of TIGAR modulation on the interaction of cancer cells with the stromal cells of the TME, we examined PDAC tumors from both KFC-KO and KPC-Tg mice. PDAC are characterized by extensive desmoplastic stroma consisting of fibroblasts and immune cells. Using trichrome staining to detect collagen, we noted a clear increase in KFC-KO PDAC tumors (Fig. 2*A*) that was evident in at both early and late stages of tumor development (Fig. 2*B*). The KFC-KO tumors also showed an increase in CAFs, detected by staining for alpha smooth muscle actin (⍺SMA, Fig. 2*C*) and fibroblast activation protein (FAP, Fig. 2*D*). KPC-Tg tumors that overexpress TIGAR showed a reduction in both ⍺SMA and FAP staining (*SI Appendix*, Fig. S2 *A* and B). Taken together, these results suggested that modulation of TIGAR expression in the cancer cells (resulting in increased or decreased ROS) could control the accumulation of tumor-promoting fibroblasts. To examine whether this was a direct consequence of the tumor/fibroblast interaction, we moved to an in vitro system, exposing primary mouse embryonic fibroblasts to conditioned medium from KFC or KFC-KO cell lines (Fig. 2*E*). ⍺SMA expression was low in these normal, untransformed fibroblasts and this was not clearly influenced by exposure to conditioned medium from control KFC cell lines. However, fibroblasts showed a clear increase in ⍺SMA expression after exposure to medium conditioned by KFC-KO cells, an effect that was also seen in immortalized NIH3T3 fibroblasts (*SI Appendix*, Fig. S2*C*). Importantly, treatment of the KFC-KO cells with the antioxidant NAC diminished their ability to condition medium to drive the reprogramming of fibroblasts (Fig. 2*E*). To investigate whether fibroblasts exposed to KFC-KO cells would have a greater influence on the migratory ability of PDAC cells compared to fibroblasts exposed to KFC cells, we tested the effect of including fibroblasts on cancer cell migration. While the inclusion of mouse embryonic fibroblasts has only a modest effect on the migratory capacity of control KFC cells, the migratory capacity of KFC-KO cells (which was already higher than control KFC cells) was dramatically increased in the presence of fibroblasts (Fig. 2*F*).

**Fig. 3.**
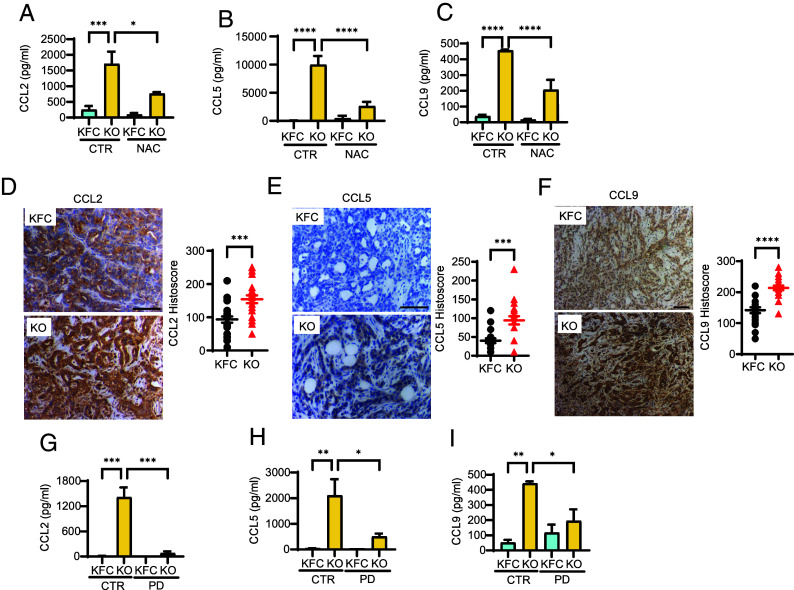
Cytokines secretion by TIGAR deficient PDAC cells. (*A*–*C*) ELISAs of (A) CCL2, (*B*) CCL5, and (*C*) CCL9 in media collected from KFC and KFC-KO cell lines with (NAC) and without (CTR) NAC treatment. (*D*–*F*) Immunohistochemistry and quantification of (*D*) CCL2, (*E*) CCL5, and (*F*) CCL9 expression in PDAC tumors from KFC and KFC-KO mice. Each datapoint represents values obtained from an individual animal. n = 21-26. (*G*–*I*) ELISAs of (*G*) CCL2 (*H*) CCL5 (*I*) CCL9 from media collected from KFC and KFC-KO cell lines with (PD) and without (CTR) the ERK inhibitor PD98059 (50 µM). Data in (*A*–*C* and *G*–*I*) were analyzed by one-way ANOVA with Tukey post hoc test, n = 3 independent KFC and KFC-KO cell lines. Error bars represent mean ± SEM. Data in (*D*–*F*) were analyzed by two-tailed Student’s t test. Error bars represent mean ± SEM. * *P* < 0.05; ** *P* < 0.01; *** *P* < 0.001; **** *P* < 0.0001. (Scale bar, 100 μm.)

The ability of conditioned medium from the PDAC cells to influence fibroblasts suggested that the cancer cells were secreting a factor that was modulated by the presence or absence of TIGAR. Analysis of cytokine arrays exposed to conditioned medium derived from KFC and KFC-KO cells revealed an increase in several cytokines in the medium from KFC-KO cells that was limited by treatment of the cells with the antioxidant NAC (*SI Appendix*, Fig. S3*A*). Subsequent ELISAs confirmed the increased secretion of CCL2, CCL5, and CCL9 from KFC-KO cells, which was significantly limited by treatment of the cells with NAC (Fig. 3 *A*–*C*). Higher levels of CCL2, CCL5, and CCL9 were also seen in KFC-KO compared to KFC PDAC tumors (Fig. 3 *D*–*F*). KPC-Tg cells showed a significant decrease in secretion of CCL2, CCL9, and CCL5 (*SI Appendix*, Fig. S3 *B*–D), consistent with the changes seen in response to TIGAR deletion. KPC-Tg PDAC tumors also showed a decreased expression of these three cytokines (*SI Appendix*, Fig. S3 *E*–G). Interestingly, the increase in cytokine secretion in KFC-KO cells was almost completely eliminated by treatment with an ERK inhibitor PD98059 (Fig. 3 *G*–*I*), indicating a role for increased MAPK signaling in response to increased ROS in driving this response.

**Fig. 4.**
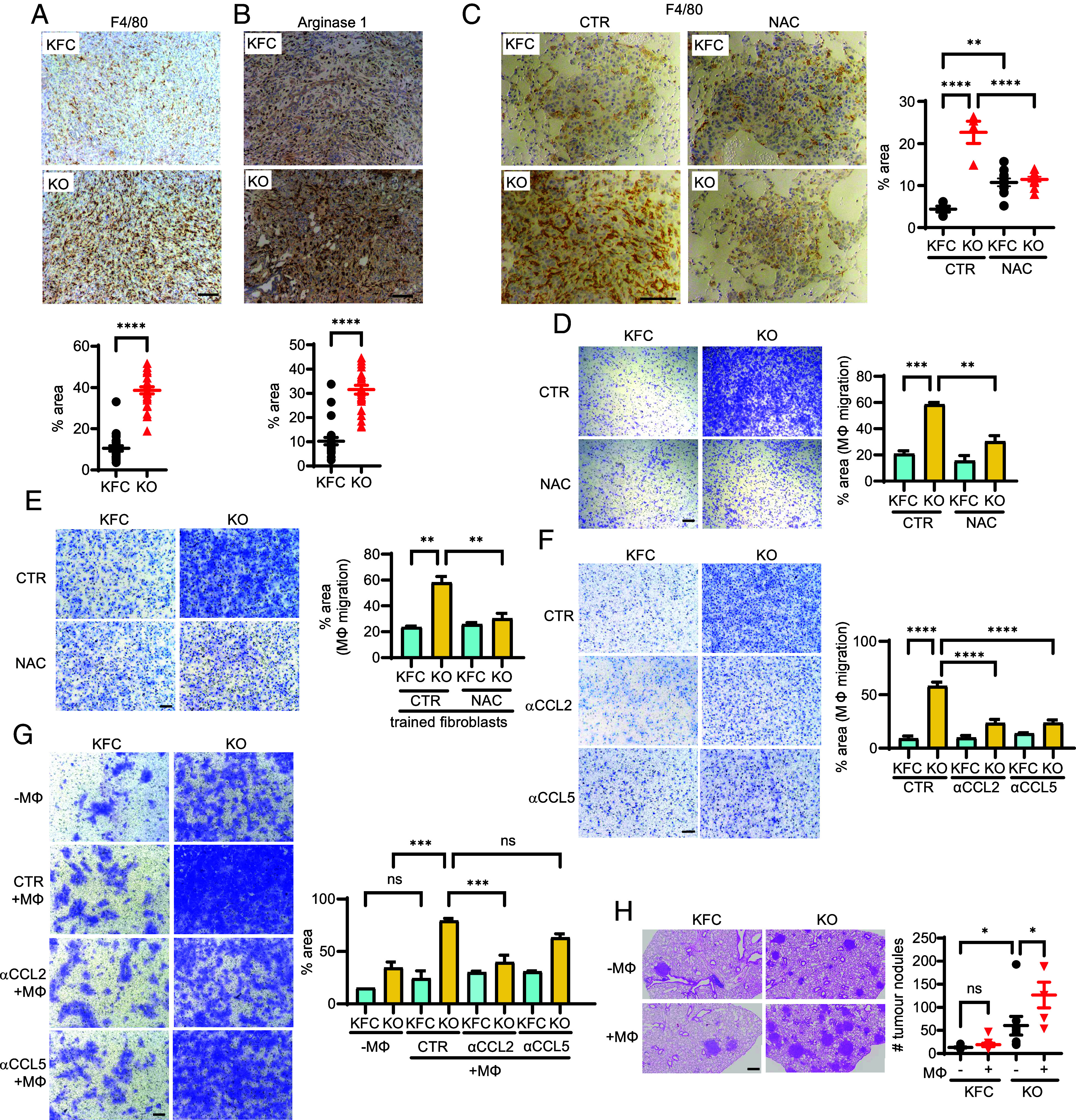
TIGAR-deficient PDAC cells modulate macrophages, further increasing migration and invasiveness. (*A* and *B*) Immunohistochemistry and quantification of (*A*) F4/80 (*B*) Arginase 1 expression in PDAC from KFC and KFC-KO mice. (*C*) Immunohistochemistry and quantification of F4/80 staining in lung metastasis from tail vein injection of KFC and KFC-KO cells, with (NAC) and without NAC (CTR) treatment. Each data point represents values obtained from an individual mouse. CTR treated KFC and KFC-KO n = 4, NAC treated KFC and KFC-KO n = 10. (*D*) Migration of macrophages (Mϕ) toward KFC and KFC-KO cells with (NAC) and without (CTR) NAC treatment. (*E*) Migration of macrophages (Mϕ) toward fibroblasts cocultured with KFC or KFC-KO cell lines with (NAC) and without (CTR) NAC treatment. (*F*) Macrophage (Mϕ) migration toward KFC and KFC-KO cells with and without CCL2 and CCL5 neutralizing antibodies. (*G*) Migration of KFC and KFC-KO cells with (+Mϕ) or without (−Mϕ) macrophages, with (⍺CCL2, ⍺CCL5) and without (CTR) CCL2 and CCL5 neutralizing antibodies. (*H*) Lung metastasis from tail vein injected KFC and KFC-KO cell lines previously cocultured with (+Mϕ) or without (−Mϕ) macrophages. Each data point represents values obtained from an individual mouse (−Mϕ KFC n = 10; + Mϕ KFC n = 9; - Mϕ KFC-KO n = 8; + Mϕ KFC-KO n = 5). Data in (*A* and *B*) were analyzed by two-tailed Student’s t test. Error bars represent mean ± SEM. Data in (*C*–*H*) were analyzed by one-way ANOVA with Tukey post hoc test. Error bars represent mean ± SEM. (*A* and *B*) Each data point represents values obtained from an individual mouse. n = 23-24. (*D-G*) n = 3 independent KFC and KFC-KO cell lines. *P < 0.05; **P < 0.01; ***P < 0.001; ****P < 0.0001. (Scale bar, 100 μm.)

Numerous studies have shown a role for cancer cell–derived cytokines in promoting the accumulation of tumor supporting macrophages, characterized by their production of Arginase 1 (14). KFC-KO tumors showed an increase in F4/80 staining myeloid cells (Fig. 4*A*) with enhanced staining for Arginase 1 (Fig. 4*B*), consistent with the presence of tumor-supportive macrophages (34). KPC-Tg tumors showed decreased staining for these cells (*SI Appendix*, Fig. S4*A*) with less expression of Arginase 1 (*SI Appendix*, Fig. S4*B*). Interestingly, KFC-KO lung metastases also contained more myeloid cells than TIGAR-expressing KFC metastases, but this effect was lost in mice treated with NAC (Fig. 4*C*). Previous work has shown that macrophages are the predominant myeloid cell population in PDAC, and we focused on understanding the consequences of TIGAR loss on the PDAC/macrophage interaction. In vitro analyses showed that KFC-KO cells prompted a significant increase in bone marrow derived–macrophage migration (Fig. 4*D*). The ability of NAC to limit this response (Fig. 4*D*) mirrored the ability of NAC to limit the excretion of cytokines from these cancer cells (Fig. 3*A*). TIGAR overexpressing KPC-Tg tumor cells showed a lower ability to attract macrophages compared to KPC cells (*SI Appendix*, Fig. S4*C*). Furthermore, fibroblasts that had been co-cultured with KFC-KO PDAC cells were able to promote a significant increase in bone marrow–derived macrophage migration compared to the fibroblasts that were cultured with KFC cells (Fig. 4*E*) and activity that was also limited by NAC. These data suggest that the increased infiltration of macrophages in KFC-KO PDAC tumors reflects both a direct response to cytokine secretion by the tumor cells combined with a function for tumor-educated fibroblasts.

The results are consistent with an ability of ROS-induced cytokine secretion from PDAC cells to attract tumor-supporting macrophages. To examine directly whether the production of cytokines by the KFC-KO cells is causing increased macrophage migration, we carried out the analysis in the presence of neutralizing antibodies. Neutralizing antibodies against CCL2 and CCL5 lowered the ability of the KFC-KO cells to attract macrophages (Fig. 4*F*), while antibodies against CCL9 did not have any significant effect on the migration of macrophages (*SI Appendix*, Fig. S4*D*). Previous studies have shown that tumor-derived CCL2 and CCL5 can attract macrophages that enhance the metastatic capacity of the primary tumor (35–37). Using coculture of macrophages with PDAC cells, we were unable to detect a clear effect of macrophages on the migration of KFC cells. By contrast, the presence of macrophages further enhanced the migration of the KFC-KO cells, an effect that was decreased by neutralizing CCL2 (Fig. 4*G*). Neutralization of CCL5 or CCL9 did not significantly decrease the ability of macrophages to enhance PDAC cell migration (Fig. 4*G* and *SI Appendix*, Fig. S4*E*). To test whether this impact of macrophages on PDAC cells was also seen in vivo, we cocultured KFC-KO and control KFC cells with bone marrow–derived macrophages before performing tail vein injection of these PDAC cells in mice, comparing lung colonization capacity with PDAC cells that had not been pre-exposed to macrophages (Fig. 4*H*). In concordance with the migration assays, the lung colonization by WT PDAC cells was not significantly impacted by the presence of macrophages. As seen previously, KFC-KO cells formed more lung colonies than KFC cells, and the coculture with macrophages before the tail vein injection further enhanced the invasiveness of these TIGAR null cells (Fig. 4*H*).

## Discussion

Our previous work showed fluctuations in the level of TIGAR expression during the development of PDAC in both mouse and human systems, with lower expression in the invasive tumors correlating with increased metastatic capacity (4). Here, we show that engineered increase in TIGAR expression in the pancreas can also contribute to the modulation of the metastatic potential of PDAC cells. In the less metastatic model (KFC, driven by KRAS mutation and loss of p53), loss of TIGAR increased the incidence of lung metastasis, while in the more aggressive model (KPC, driven by mutation in KRAS and mutation in p53), enhanced TIGAR expression decreased lung metastasis. While these responses to less or more ROS are consistent, we would predict that increased ROS due to TIGAR deficiency in the KPC model may further enhance mutant p53’s ability to increase invasiveness and desmoplasia, due to the previously reported observation that increase of ROS can stabilize mutant p53 (38). Liver metastases were not affected by changes in TIGAR expression in either model, indicating that the ROS-dependent effect of TIGAR modulation is tissue dependent. Future work is required to understand the mechanism underlying how increased ROS in PDAC cells makes them selectively more capable of forming lung metastases.

Loss of TIGAR and increased ROS was previously shown to drive changes in PDAC that resulted in an increased epithelial to mesenchymal transition and enhanced ERK signaling resulting from decreased DUSP6 expression (4). Here, we see that each of these responses is lower in TIGAR overexpressing PDAC cells. While these cell intrinsic changes can control migration and metastatic capacity, we now show that loss of TIGAR and increased ROS in PDAC cells also profoundly affect the cells of the TME. Our results indicate that while KFC-KO cells alone are more migratory than KFC cells, the lack of TIGAR in these tumors also modulates the TME via cytokine secretion, mobilizing stromal cells such as fibroblasts and macrophages to further enhance the migratory and metastatic capacity of these cells. Pancreatic stellate cells (PSCs) represent only a minor contribution to CAFs, which are mostly non-PSC-derived. However, both CAF populations express high ⍺SMA (39) and our observation of a general increase of ⍺SMA in the KFC-KO tumors suggests that both PSC-derived and non-PSC-derived cells are affected. A more in-depth analysis of the different cell populations will determine whether increased ROS following TIGAR deletion has differential effects on modulating the ability of these different CAFs to control metastasis. Our observation that increased CAFs in the TIGAR deficient tumors influences the activity and recruitment of macrophages is in agreement with previous studies showing the role of CAF-dependent recruitment and polarization of macrophages in promoting tumor invasiveness in PDAC (40), colorectal cancer (41), and breast cancer models (42). A role for increased oxidative stress in the promotion of noncell autonomous, proinvasive effects by inducing paracrine secretion of proinvasive factors has previously been shown in untransformed mammary cells (6). It is well established that tumor secreting cytokines such as CCL2 and CCL5 attract macrophages polarized to support cancer cell dissemination (35–37, 43–50), and consistently we find increased levels of Arginase 1 (as a marker of these myeloid cells in neoplastic pancreas) in TIGAR null primary tumors and metastases. However, we also see that when PDAC cells are experimentally exposed to equal numbers of macrophages, the impact on migration and lung colonization of KFC-KO cells remains substantially higher than that seen with KFC cells. Interestingly this activity of macrophages is limited by neutralizing CCL2, suggesting that CCL2 (produced by KFC-KO PDAC cells) can promote the capacity of macrophages to enhance migration of the tumor cells through mechanisms that are additional to the ability of CCL2 to attract macrophages into the tumor. This likely reflects the well-established observation that cancer cell–induced changes in macrophage behavior, not just numbers, contribute to protumorigenic activity of these cells (51). These data suggest that there is a synergy between cell-intrinsic and cell-extrinsic effects of loss of TIGAR in promoting metastatic capacity.

The impact of increased cancer cell ROS on metastasis is tumor type dependent. In melanoma and lung cancer, loss of the ability to limit ROS through antioxidant defense has been shown to decrease metastasis due to the induction of ROS-dependent death in the cancer cells (7, 52–54). By contrast other tumor systems—such as the PDAC model described in this study—show an increase in metastatic potential in response to elevated ROS (5, 55–58). Intriguingly, while the antioxidant NAC reduced the infiltration of macrophages into KFC-KO lung deposits in the experimental metastasis model, we found that NAC treatment significantly increased macrophage infiltration in KFC lung colonies (Fig. 4*C*). This observation mirrors previous work showing that NAC decreases the ability of KFC-KO cells to form lung colonies but increases this capacity of wild-type KFC cells (4). The complexity of these responses may reflect different contributions of ROS at different stages of the metastatic process or other alterations in the interaction between TIGAR null tumor cells and different components of the noncancer stromal compartments that may selectively mitigate against the toxic effect of elevated ROS.

## Materials and Methods

### In Vivo Animal Studies.

All animal studies adhered to the UK Animals (Scientific Procedures) Act 1986 (Project License 70/8645, P319AE968) and EU Directive 2010, with ethical approval from the University of Glasgow and The Francis Crick Institute. Mice were maintained in a pathogen-free environment under controlled conditions (temperature 19 to 23 °C, humidity 55 ± 10%, 12-h light/dark cycle) with unrestricted access to food and water. A minimum acclimatization period of 2 d (on-site bred) or 7 d (imported) was implemented prior to experimentation. Mice were randomly allocated to experimental groups. Both male and female mice were used in PDAC genetic models of disease (GEMMs), while only female mice were employed in the tail vein lung colonization model, which used cell lines from female mice.

### Transgenic Mouse Models for PDAC.

*Trp53^LSL-R172H/+^, Kras^LSL-G12D/+^, Trp53^fl/+^*, *Pdx1-Cre*, and *p48-Cre* strains (31, 59, 60) were interbred to obtain KFC (*Pdx1-Cre*; *Kras^LSL-G12D/+^;Trp53^fl/+^*) and KPC (*p48-Cre, Kras^LSL-G12D/+^, Trp53^LSL-R172H/+^*) mice. To introduce TIGAR deficiency in these models, *Tigar^fl/fl^* strain (61) was used to breed into the KFC to obtain *Tigar^fl/+^* or *Tigar^+/+^* for control (KFC) and *Tigar^fl/fl^* for *Tigar* knockout (KFC-KO) in a mixed background (4). To introduce TIGAR overexpression into the KPC model, the *Tigar* overexpressing allele (Tg, see below for details) was used to breed into KPC to obtain control (KPC) and TIGAR overexpressing (KPC-Tg) mice in C57BL/6 J background. Mice were monitored twice weekly and tissues were collected at the indicted time points or when exhibiting symptoms of PDAC (59). Tumor and metastatic burden were assessed by gross pathology and histology. Animals succumbing to non-tumor-related illnesses were considered censored data points. Efforts were made to maintain balanced sex ratios across groups to minimize gender-based variability. Mice were randomly allocated to treatment groups. Data collection was performed in a blinded manner.

### TIGAR Overexpressing Allele.

A targeting vector was constructed to allow the insertion of a lox–stop–lox *Tigar* cDNA at the mouse Hprt locus. To achieve this, a mouse *Tigar* cDNA sequence was first cloned by PCR using oligos tailed for cloning into the NheI and XhoI sites of pHprtCAGSA.STOP (62). The elements containing the CAGSA-STOP-TIGARcDNA-pA were then inserted into the Hprt-targeting vector pSKB1 (63) by recombining in DY380 *Escherichia coli* (64). HM1 mouse embryonic stem cells [mESCs; (65)], which carry a deletion at the Hprt locus, were transfected with the *Tigar* cDNA targeting vector using standard techniques. Following transfection, the mESCs were cultured in HAT medium to select for cells that had regained Hprt activity. Surviving clones of mESCs were picked and genomic DNA from the clones was used to screen for insertion of the *Tigar* cDNA. Correct insertion of the targeting vector at the Hprt locus was confirmed by long-range PCR across both the 5’ and 3’ homology arms (Expand Long Template PCR System, Roche). The primers used for genotyping targeted ES cells were at the 5′ prime side: 5’ GTTGCTGAGGCAAAAATAGTGTAAT 3’ and 5’ CCATTTACCGTAAGTTATGTAACGC 3’ and at the 3’ side: 5′ CTACCTAGTGAGCCTGCAAACTG 3’ and 5’ ATGTAAGTGCTAGGAATTGAACCTG 3’.

Following the identification of correctly targeted clones for the *Hprt-LSL-Tigar* allele, mouse lines were subsequently derived by microinjection of targeted mESCs cells into C57BL/6 J blastocysts according to standard protocols (Nagy et al. 2003). After breeding of chimeras, germline offspring were identified by coat color and the presence of the modified allele was confirmed with PCR using oligos specific for the *Tigar* cDNA and polyadenylation sequence: 5’ TCCTCCATCACTCCCAACAC 3’ and 5’ GGTTCTTTCCGCCTCAGAAG 3’. Following successful validation of the mouse strain carrying the *Hprt-LSL-Tigar* targeted allele, genotyping was subsequently carried out by a commercial genotyping service provider (Transnetyx, Cordova, TN).

### Lung Metastasis Model.

2 × 10^5^ KFC and KFC-KO cells per mouse in 100μL PBS were injected (tail vein) into athymic nu/nu mice (females, The Jackson Laboratory, Bar Harbor, ME). Lung tissue was harvested for histological examination 14 d postinjection. To assess the effects of antioxidant treatment, mice received N-acetyl-L-cysteine (NAC, Sigma A7250) in drinking water (1 g/L, pH 7) for 1 wk prior to and throughout the experimental period. PDAC cells were exposed to NAC overnight before trypsinization and subsequent injection into mice. For coculture experiments, PDAC cells from control (KFC) and TIGAR deleted KFC-KO mice were cocultured with bone marrow–derived macrophages for 3 d (cells without coculture were used as control), and then, 1 × 10^5^ PDAC cells per mouse in 100μL PBS were injected (tail vein) into athymic nu/nu. After 14 d, lung tissues were collected for histological analysis.

### Cell Cultures and Treatments.

Tumor cell lines were derived from control (KPC, n = 3) and TIGAR overexpressing (KPC-Tg, n = 4) tumors as previously described (4). KFC and KFC-KO cell lines have been described previously (n = 3 KFC and KFC-KO lines) (4). Briefly, tumor tissue samples were collected in phosphate-buffered saline supplemented with penicillin-streptomycin and subsequently minced into small fragments. Enzymatic digestion was performed using a mixture of collagenase type I (200 U/mL, Gibco) and dispase (2.4 U/mL, Gibco) in Hank's Balanced Salt Solution for 1 h at 37 °C to liberate individual cells. Following two washes with HBSS, the cell pellet was resuspended and cultured in Dulbecco's Modified Eagle Medium enriched with 10% fetal bovine serum, 2 mM L-glutamine, and penicillin-streptomycin. To obtain macrophages, bone marrow was extracted from the tibia and femur of 8 to 10-wk-old C57Bl/6J mice using phosphate-buffered saline (PBS). Red blood cells were eliminated through lysis with a 10× RBC lysis buffer (BioLegend, cat. no. 420301). Subsequently, 5 × 10^6^ bone marrow cells were cultured in nontissue culture-treated plates containing 20 ng/mL M-CSF (Peprotech #315-02) to induce macrophage differentiation. PD98059 (PD, Tocris cat# 1213) and N-acetyl-L-cysteine (NAC, Sigma A7250) were used at the indicated times and concentration. Neutralizing antibodies used: CCL2 (500 ng/mL, Novus Biologicals NBP1-07035), CCL5 (500 ng/mL, R&D Systems MAB478-100), CCL9 (1 µg/mL, R&D Systems AF463). In vitro experiments were performed using separate independent PDAC cell lines, or treatment of fibroblasts/macrophages with separate independent PDAC cell lines, with three independent experiments in triplicate carried out in each case.

### Transwell Migration Assays.

Transwell migration assays were conducted using Corning® BioCoat™ Control Inserts with 8 µm pore size for PDAC cells and 5 µm pore size for macrophages. Cells were pretreated overnight with 1% serum in the presence or absence of experimental drugs. On the following day, cells were seeded into the upper chamber of the transwell insert, continuing drug treatment. The lower chamber contained media supplemented with 10% serum, with or without drug treatment. For coculture experiments, macrophages or mouse embryonic fibroblasts were seeded in the lower chamber for PDAC migration assays, and PDAC cells were seeded in the lower chamber for macrophage migration assays. After 16 h, cells on the upper membrane surface were removed, and migrated cells on the lower surface were fixed with 70% ethanol and stained with 0.5% crystal violet. Cell migration was quantified by measuring the stained area using ImageJ software and expressed as a percentage of the total area.

### Wound Scratch Assay.

A confluent cell monolayer was subjected to a scratch wound by a P20 pipette tip. Cellular debris was removed by gentle washing with 2 × complete culture medium. Images were captured at the assay initiation and after 16 h using a phase contrast microscope. Wound width was quantified through ImageJ analysis, with wound closure expressed as a percentage of initial wound area.

### Histology and Immunohistochemistry.

Histology and immunohistochemistry were performed as previously described (4). Briefly, tissue samples were preserved in 10% neutral-buffered formalin and subsequently embedded in paraffin for histological processing. Antigen retrieval was achieved through heat-induced epitope retrieval using sodium citrate buffer (Vector Laboratories, H-3300). To minimize background staining, endogenous peroxidase and avidin/biotin activity were blocked (Vector Laboratories, SP-6000) prior to immunostaining. Primary antibody incubation was performed overnight at 4 °C in a solution containing 10% normal horse serum and 1 × TBST. For immunohistochemistry, primary antibodies used were anti-MDA (1:300, Abcam Ab6463), anti-phospho-ERK (1:600, Cell Signaling Technology), anti-DUSP6 (1:300, Abcam Ab76310), anti-E-Cadherin (1:300, Cell Signaling Technology), anti-⍺SMA (1:600, Abcam Ab5694), anti-FAP (1:600, Abcam Ab53066), anti-CCL2 (1:600, Novus Biologicals NBP1-07035), anti-CCL5 (1:600, R&D Systems MAB478-100), anti-CCL9 (1:600, R&D Systems AF463), anti-F4/80 (1:1,000, Cell Signaling Technology 70,076), anti-Arginase1 (1:600, Invitrogen PA5-85267), and anti-cytokeratin 19 (CK19) (1:600, Abcam Ab52625). Expression levels of proteins were scored based on staining intensity and area of tumor cells using a weighted histoscore calculated from the sum of (1 × % weak staining) + (2 × % moderate staining) + (3 × % strong staining). For staining for cell types such as macrophages and fibroblasts, percentage area in PDAC tumors that showed positive staining was quantified using ImageJ to quantify stained area. Each data point represents values obtained from an individual mouse.

### Western Blot Analysis, ELISA, and Cytokine Arrays.

Western blot analyses were carried out as previously described (4). Briefly, total protein lysates were prepared using RIPA buffer (Millipore) supplemented with protease (Roche) and phosphatase inhibitors (Thermo Fisher Scientific). Protein concentrations were determined using the Bradford assay (QuickStart Bradford 1 × Dye Reagent) and samples were normalized accordingly. Proteins were resolved by SDS-PAGE on a 4 to 12% Bis-Tris gradient gel (Invitrogen), transferred to a nitrocellulose membrane and detected with appropriate horseradish peroxidase-conjugated secondary antibodies and ECL chemiluminescence kits (Pierce). Representative blots from at least three independent experiments are shown. Antibodies used are anti-Lamin A/C (1:1,000, Cell Signaling Technology #2032), anti-mtHSP70 (1:1,000, Invitrogen MA3-028), anti-TIGAR (1:1,000, Santa Cruz sc-67273), anti-p-ERK1/2 Thr202/204 (1:1,000, Cell Signaling Technology), anti-DUSP6 (1: 500 Abcam Ab76310), anti-E-Cadherin (1:5,000, Cell Signaling Technology #3195), anti-Actin (1:10,000, Abcam Ab20272), anti-ERK (1:1,000, Cell Signaling Technology #9102), and anti-⍺SMA (1:1,000, Abcam Ab5694). ELISA for mouse CCL2 (R&D Systems DY479-05), CCL5 (R&D Systems DY478-05), and CCL9 (R&D Systems DY463) were performed according to the manufacturer’s instructions. Cytokine Antibody Array (RayBiotech, C2000) was performed according to the manufacturer’s instructions.

### Quantification and Statistical Analysis.

Data were analyzed using GraphPad Prism 10 software (GraphPad Software). The survival data were analyzed by log-rank Mantel–Cox test. Fisher’s exact test was used to compare the frequency of metastasis. Other data represent mean values ± SEM from at least three independent experiments (n ≥ 3). Student’s t test (comparisons between two groups) and one-way ANOVA with Tukey post hoc (comparisons of three or more groups with one independent variable) were used as indicated in the legends.

## Supplementary Material

Appendix 01 (PDF)

Dataset S01 (XLSX)

## Data Availability

All study data are included in the article and/or *SI Appendix*.
