# Supplementary material for: Regulation of ROS signaling by TIGAR induces cancer-modulating responses in the tumor microenvironment
Source: Proc Natl Acad Sci U S A. 2024 Dec 5;121(50):e2416076121. doi: 10.1073/pnas.2416076121 (PMC11648893; doi:10.1073/pnas.2416076121)
Supplement: Supplementary file 1 — Appendix 01 (PDF) [file pnas.2416076121.sapp.pdf]

Supporting Information for

Regulation of ROS signaling by TIGAR induces cancer-modulating responses in the tumor microenvironment

Eric C Cheung[1*] Douglas Strathdee[2], David Stevenson[2], Jack Coomes[1], Karen Blyth[2,3] and Karen H Vousden[1*]

Eric.cheung@crick.ac.uk
Karen.vousden@crick.ac.uk

This PDF file includes:

Figures S1 to S4

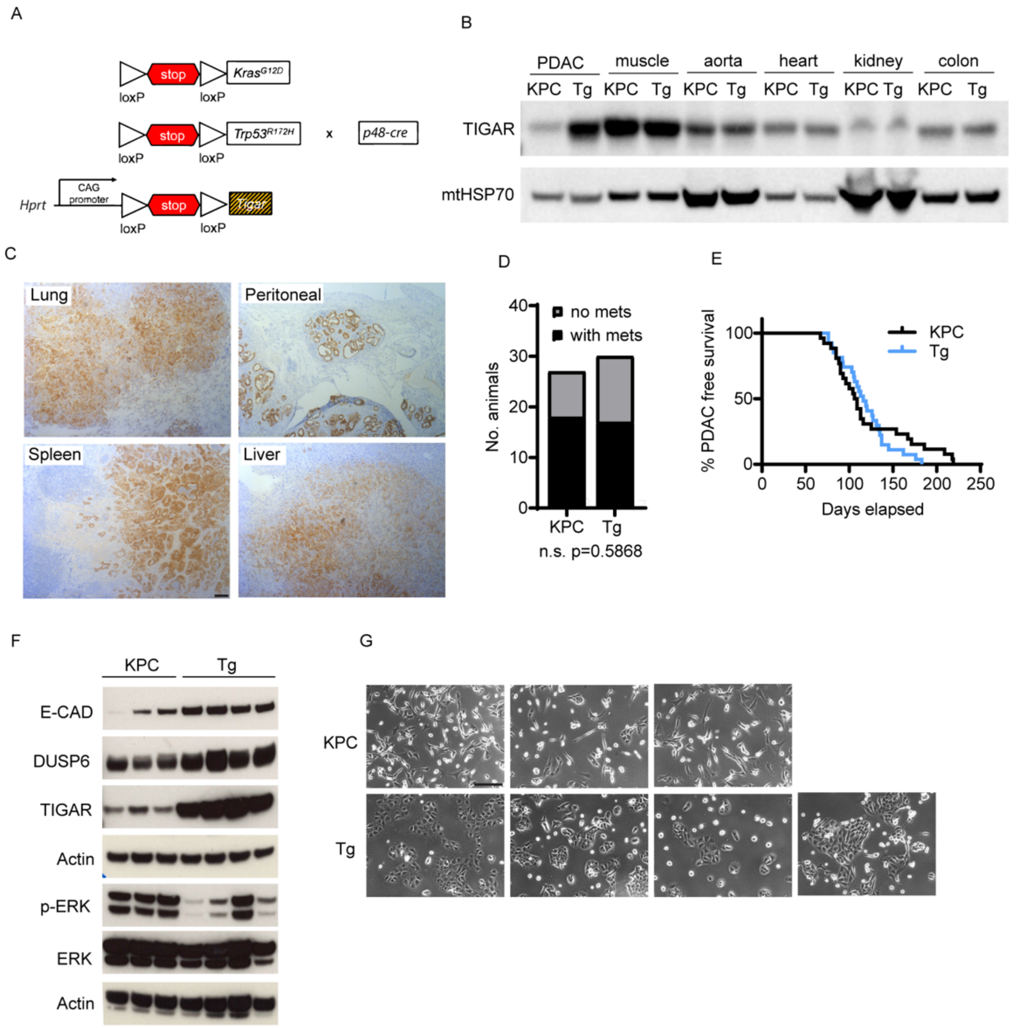

SI Figure 1 (related to Figure 1).

(A) Schematic of mouse PDAC model with TIGAR overexpression (KPC-Tg). (B) Western blot analysis of TIGAR in organs isolated from KPC and KPC-Tg mice. Note at this point (around 6 months) the pancreas of these mice is predominantly PDAC. Representative of n=3 mice. Loading control is mtHSP70. (C) Examples of metastasis in KPC-Tg mice. Representative of n=30 mice. CK-19 staining indicates the metastatic PDAC cells. (D) Number of mice with metastasis in KPC and KPC-Tg mice. (E) Kaplan-Meier tumor free survival curve of KPC and KPC-Tg mice. (F) Western blot analysis of PDAC cell lines isolated from KPC and KPC-Tg PDAC tissues. n=3 independent cell lines for KPC and n=4 independent cell lines for KPC-Tg. Loading control is Actin. (G) Morphology of cells isolated from KPC and KPC-Tg PDAC. n=3 independent cell lines for KPC and n=4 independent cell lines for KPC-Tg. Data in (D) were analyzed by Fisher's exact test. KPC: n=27, KPC-Tg: n=30. Data in (E) were analyzed by log rank test. Scale bar, 100µm.

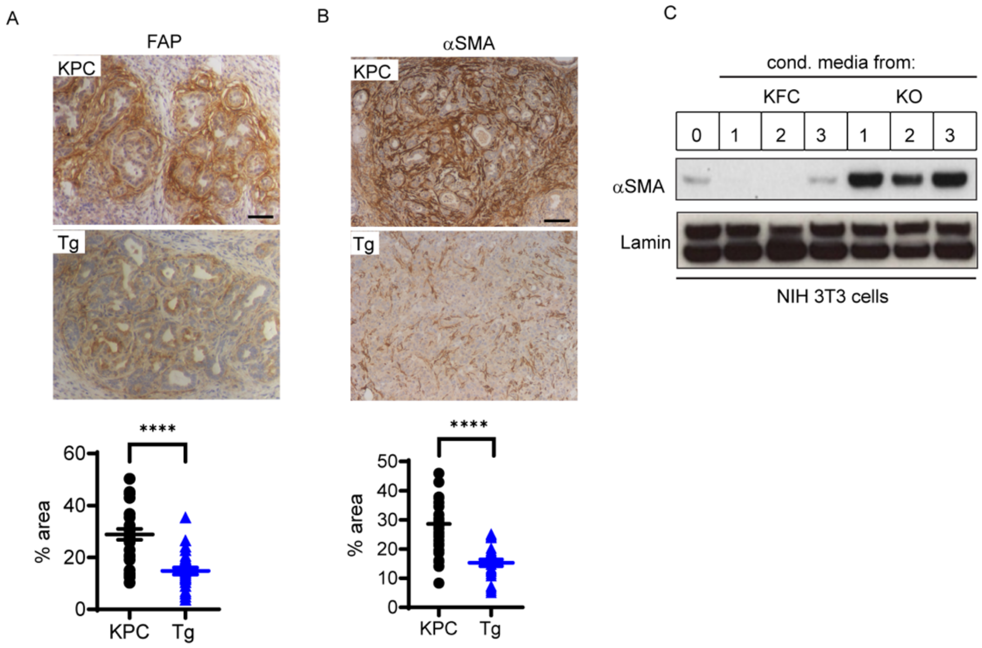

SI Figure 2 (related to Figure 2).

(A, B) Immunohistochemistry and the quantification of (A) FAP and (B) αSMA expression in KPC and KPC-Tg mice. (C) Western blot analysis of αSMA level in NIH 3T3 cells co-cultured with KFC and KFC-KO cell lines. Loading control is Lamin. Data in (A, B) were analyzed by two-tailed Student's t test. Each data point represents values obtained from an individual animal. n=27-30. Error bars represent mean ± SEM. Scale bar, 100 µm. * $P < 0.05$; ** $P < 0.01$; *** $P < 0.001$; **** $P < 0.0001$. Scale bar, 100 µm.

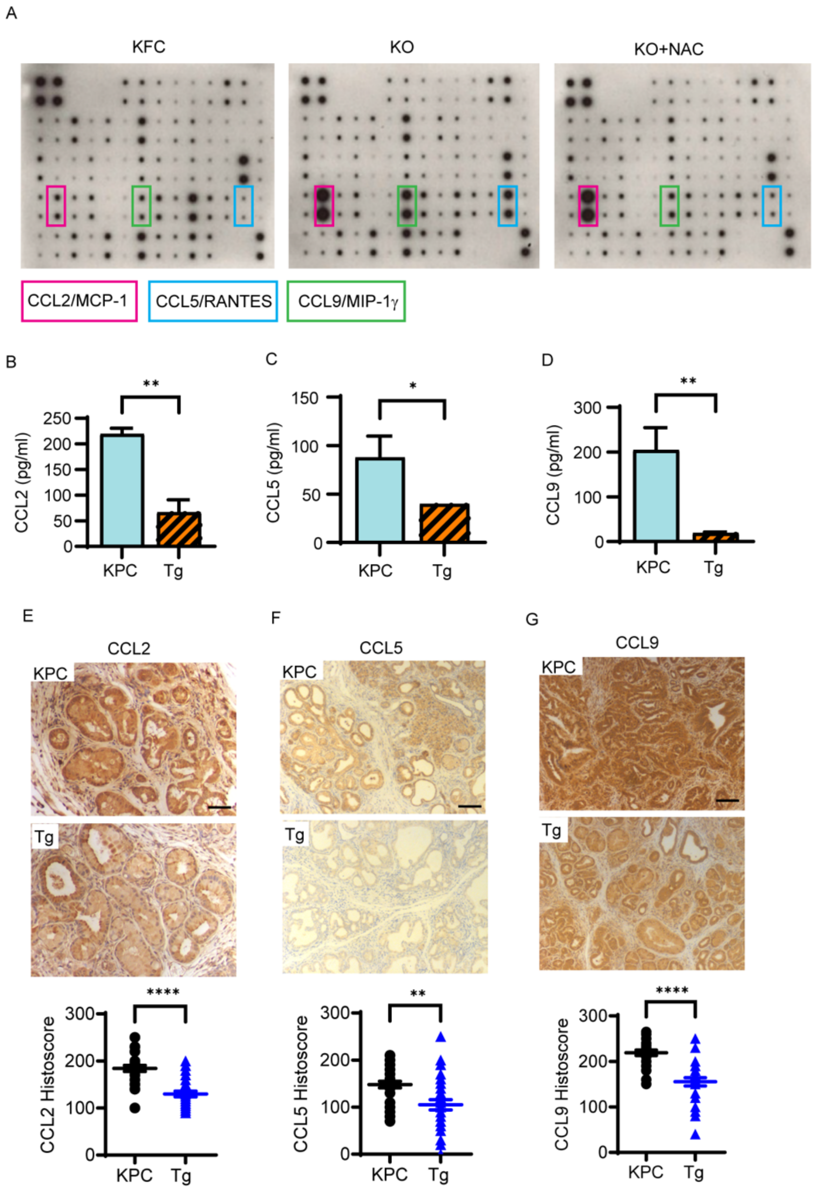

SI Figure 3 (related to Figure 3).

(A) Cytokine array of media collected from KFC, KFC-KO and KFC-KO cell lines treated with NAC (KO+NAC). (B-D) ELISA assays of (B) CCL2 (C) CCL5 (D) CCL9 in media collected from KPC and KPC-Tg cell lines. (E-G) Immunohistochemistry and quantification of (E) CCL2 (F) CCL5 (G) CCL9 expression in PDAC tumors from KPC and KPC-Tg mice. (B-D) n=3 independent KPC and n= 4 independent KPC-Tg cell lines. (E-G) Each data point represents values obtained from an individual mouse. n=27-30. Data in (B-G) were analyzed by two-tailed Student's t test. Error bars represent mean ± SEM. *P < 0.05; ** P < 0.01; *** P < 0.001; **** P < 0.0001. Scale bar, 100μm**.**

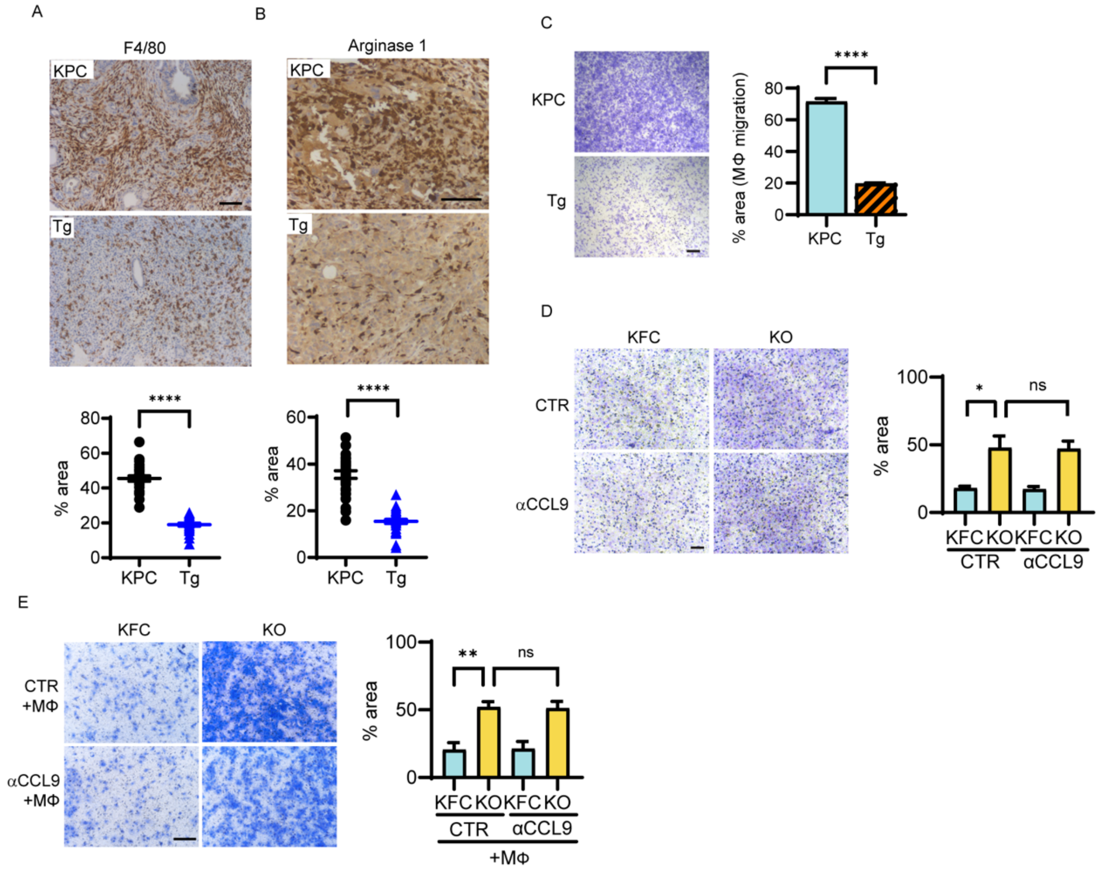

SI Figure 4 (related to Figure 4).

(A, B) Immunohistochemistry and quantification of (A) F4/80 and (B) Arginase 1 expression in PDAC from KPC and KPC-Tg mice. (C) Migration of macrophages (Mϕ) towards KPC and KPC-Tg cells (D) Migration of macrophages (Mϕ) towards KFC and KFC-KO cells with (αCCL9) and without (CTR) CCL9 neutralizing antibodies. (E) Migration of KFC and KFC-KO cells towards macrophages (+Mϕ) with (αCCL9) and without (CTR) CCL9 neutralizing antibodies. Data in (A-C) were analyzed by two-tailed Student's t test. Error bars represent mean ± SEM. Data in (D, E) were analyzed by one-way ANOVA with Tukey post hoc test. Error bars represent mean ± SEM. (A, B) Each data point represents values obtained from an individual mouse. n=27-30 (C) n=3 independent KPC and n= 4 independent KPC-Tg cell lines (D, E) n=3 independent KFC and KFC-KO cell lines. * $P < 0.05$; ** $P < 0.01$; *** $P < 0.001$; **** $P < 0.0001$. Scale bar, 100μm.
